# Pneumococcal Pyæmia

**Published:** 1910-08-13

**Authors:** 


### PNEUMOCOCCAL PYÆMIA.

It is well enough recognised that typhoid fever is not an intestinal disease, but is a specific fever with a generalised infection, including bacteriæmia, with a local manifestation in the bowel. It is a mistake to suppose that lobar pneumonia due to the pneumococcus is a lung disease; it is really a generalised infection with a local manifestation in the lung. It is often associated with bacteriæmia without the patient presenting evidence of disease elsewhere than in the lung. That the generalised effects of the pneumococcus are more important than the local changes in the lungs that are due to it is evidenced in many ways, the two most striking of which are, first, that some patients are very ill indeed with only a small extent of pneumonic consolidation, whilst others may have double pneumonia without being very ill at all; and, secondly, by the fact that the extent of the change in the lung immediately before the crisis is practically the same as it is immediately after the crisis, and yet the general condition of the patient undergoes a very remarkable improvement in the course of a few hours. This improvement at the crisis has no corresponding change in the lung lesions being due more to the general body reaction to the pneumococcus than to anything that occurs particularly in the lungs. It is therefore surprising that generalised lesions visible to the naked eye are not commoner than they are in lobar pneumonia. That they may be numerous and widespread, however, is well known, and is well illustrated by the way in which patients may recover even from the most extensive lesions, as in the following case, reported by Dr. Letulle and Dr. Leconte at the Société Médicale de Paris. The patient was a chauffeur, forty-one years of age, who was admitted to hospital on June 21 for pneumonia, which had started the previous day with pains in the side and a severe rigor. He had for many years been addicted to drink, and he had been indulging recently. The temperature was 104° F., the urine contained a little albumin, and there were signs of right apical pneumonia. Restless delirium set in, and it was found necessary to fasten the patient to the bed. By June 25 the temperature had fallen to 102°.2 F., but on June 29 was calmer, but there were now signs of consolidation in the left lower lobe, whilst those at the right apex were diminishing. On June 26 pain developed in the left ankle, which became swollen, tender, and slightly red, while the temperature rose to 102°.2 F. Next day the temperature was 104° F.; the ankle was more swollen and painful, the redness and the swelling extending from the ankle for some distance up the leg. There being evidence of pus, an incision was made behind the external malleolus, and much thick greenish pus escaped. On June 28 the temperature had fallen to 102°.2 F., but on June 29 a large number of subcutaneous and cutaneous abscesses appeared on the back and on the left flank and over the lower part of the chest. Some of these were barely larger than smallpox pustules, whilst others were as large as bullæ. Between June 30 and July 12 these abscesses appeared in rapid succession, the largest, over the left shoulder, being 2½ inches long. The smaller ones dried up by themselves, but the larger ones had to be incised, and they got well in a few days. Altogether there were over 140 abscesses, many of which left a pigmented spot after healing. The temperature meanwhile ranged from 104° to 102° F. On July 9 the patient complained of pain on swallowing, and the thyroid gland was found to be swollen and tender. An abscess developed in the left lobe of this gland, and it had to be opened.

All the abscesses were proved to contain the pneumococcus in pure cultures, both by direct investigations and by inoculation into mice. After the opening of the thyroid abscess the patient's condition improved, and he was discharged on August 16, well in most respects, except that he still had considerable pain in his left ankle-joint when he tried to walk.

